# From Concept to Clinical Practice: Interhospital Robotic Telesurgery Using the SSI Mantra 3 Surgical Robotic System for Transabdominal Preperitoneal Repair of a Left Inguinal Hernia

**DOI:** 10.7759/cureus.99195

**Published:** 2025-12-14

**Authors:** Parimuthukumar Rajappa, Subbiah Tirunelveli Sivagnanam, Prashanth Krishna Gopalaswamy, Sabari Girieasen M, Selvaraja Venkatachalam, Shobana Mohandoss, Suprajha K Sri Sridar

**Affiliations:** 1 Institute of Robotic Surgery, Prashanth Hospitals, Chennai, IND; 2 General Surgery, Madras Medical College, Chennai, IND; 3 General Surgery, Dharan Multispeciality Hospital, Salem, IND

**Keywords:** inguinal hernia repair, inguinal hernia surgery, remote surgery, robotic-assisted surgery, robotic hernia surgery, robotic inguinal hernia repair, robotic inguinal hernia surgery, robotic surgery, robotic telesurgery, telesurgery

## Abstract

This study aimed to demonstrate and analyze the feasibility of interhospital robotic telesurgery in India using wireless networks. On August 8, 2025, a robotic telesurgery was performed between two cities in India, Chennai and Salem, using a dual-console setup of the SSI Mantra 3 robotic system (Sudhir Srivastava Innovations Pvt. Ltd., R&D HQ, Haryana, India). The robotic surgeon command center was placed in Salem, and the patient with the robotic arm cart was in Chennai. The robotic arms were docked, and a robotic transabdominal preperitoneal (TAPP) repair of the left inguinal hernia was performed via telesurgery using the SSI Mantra 3 robotic platform via a secure fiber network. The patient had an uneventful postoperative period and was discharged on postoperative day 1. This case illustrates how telesurgery can serve as a powerful tool in democratizing access to surgical expertise by eliminating the need for both patient and surgeon travel. In nations where disparities in access to advanced surgical facilities remain a challenge, the ability to deliver high-quality surgical care remotely represents a transformative step toward inclusive healthcare delivery.

## Introduction

Telesurgery, or remote surgery, is a form of telemedicine in which a surgical procedure is performed using a robotic platform, with the patient and surgeon located in separate places and connected via low-latency telecom networks [1].

Telesurgery has been successfully performed across continents, most notably in the landmark “Lindbergh operation” by Marescaux et al., which demonstrated the feasibility of remote surgery even with a mean delay of 155 ms in 2001 [1].

Two decades of progress in telecommunications have ushered in the era of wireless 5G networks, revolutionizing the field of telesurgery through their capacity for low latency and wider accessibility. This technology enables global expertise to reach underserved areas down to the last mile.

Beyond surgery, tele-mentoring, where surgeons collaborate in real time to make critical decisions, is now an immediate possibility. This case was performed using the SSI Mantra 3 Surgical Robotic System (Sudhir Srivastava Innovations Pvt. Ltd., R&D HQ, Haryana, India), the first surgical robotic system made in India. It is also the only robotic system currently approved for telesurgery in India by the Central Drugs Standard Control Organisation (CDSCO) [2].

To our knowledge, this is the first successful completion of an inter-hospital telesurgical procedure in India, conducted between Dharan Hospital, Salem, and Prashanth Hospitals, Chennai, illustrating both the technical capability and potential impact on surgical practice in the country.

## Case presentation

Patient selection

A 42-year-old male diagnosed with a left inguinal hernia was selected for robotic-assisted transabdominal preperitoneal (TAPP) hernia repair. The indication was a symptomatic hernia requiring surgical correction. The patient had no known comorbidities and a unilateral reducible inguinal hernia; he was planned for robotic TAPP hernia repair via telesurgery. Institutional Ethics Committee approval was obtained, and informed consent was provided by the patient.

Robotic setup

The surgeon command center was established at Dharan Hospital, Salem, where surgeon Dr. Parimuthukumar Rajappa operated from the console. The patient and robotic arm carts were located at Prashanth Hospitals, Chennai, where anesthesia was administered, pneumoperitoneum was created, ports were inserted, and the robotic arms were docked.

The procedure utilized the SSI Mantra 3 robotic platform, with systems installed at both Salem and Chennai, enabling synchronized inter-hospital connectivity and seamless execution. Both centers were connected to each other by audio-visual systems for seamless communication. The operating surgeon and the bedside team had a clear line of communication established to communicate during docking and instrument changes. No challenges were encountered during communication. In the event of technical issues, the Chennai team was equipped to take over and complete the procedure using the local robotic system.

Network and connectivity

**Figure 1:**
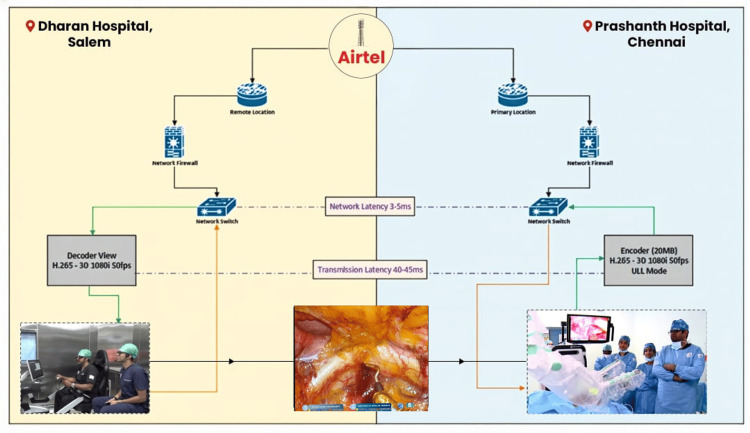
Interhospital robotic telesurgery between Salem and Chennai The figure has been created by the author, Subbiah Tirunelveli Sivagnanam.

A dedicated, secure, private fiber line by Bharti Airtel Ltd., New Delhi, India, provided the link between the two hospitals, with an average bandwidth of 50 Mbps. The connection was optimized for low-latency, real-time surgical performance, achieving a mean latency of 45 ms (range: 40-50 ms) with negligible packet loss (≤0.1%) and a jitter level ≤ 10 ms. Continuous audiovisual communication between the Salem and Chennai teams enabled precise coordination throughout the operation (Figure 1).

Results

The robotic TAPP repair of the left inguinal hernia was successfully completed on August 8, 2025, with a docking time of 13 minutes and a total console time of 124 minutes, including instrument exchanges. An atraumatic grasper and a monopolar curved scissor were used to create the peritoneal flap and complete the dissection of the myopectineal orifice.

Anatomically contoured, medium-weight polypropylene mesh was placed. The SeVana™ Suture Cutting Needle Driver (Sudhir Srivastava Innovations Pvt. Ltd., R&D HQ, Haryana, India) was used to fix the mesh to Cooper’s ligament using 2-0 polydioxanone sutures. The peritoneal flap was closed with 2-0 barbed sutures.

The mean latency during the procedure was 0.03 ms (Table 1), with uninterrupted transmission and no packet loss, allowing seamless telesurgical coordination between Salem and Chennai. No intraoperative difficulties or complications occurred, and both instrument response and image quality were maintained without delay.

**Table 1:** Network values

Network values	
Connectivity (in Mbps)	50 Mbps
Transmission latency time	40-50 ms
Data packet loss	≤ 0.1 %
Jitter service level	≤ 10 ms

The patient recovered uneventfully, was mobilized on the same day, and was discharged on postoperative day 1. At follow-up after two weeks, the surgical wounds were well-healed, and the patient had resumed normal activities without recurrence or complications.

## Discussion

Telesurgical procedures eliminate the need for robotic specialist surgeons to travel between healthcare facilities, allowing them to perform more procedures in remote areas and improving the reach of specialized care.

The Lindbergh operation was performed with a latency of 155 ms. Advances in telecommunications, the advent of 5G networks, and fiber-optic infrastructure [3] over the last two decades have enabled latency as low as 40-50 ms as in our procedure, with jitter under 10 ms. Minimal latency, well below the suggested ideal limit of 200 ms [4], allows precise dissection around delicate structures and real-time feedback to the operating surgeon at a remote command center.

With today’s commercially available telecommunications technology, telesurgery has evolved from a scientific concept into a viable and potentially lifesaving surgical approach [5]. Looking forward, widespread adoption of telesurgical platforms will also enhance remote mentoring and skill development, enabling expert surgeons to guide others in performing complex procedures through tele-mentoring, which has been shown to be as safe and effective as on-site mentoring [6].

Regional centers of excellence could be virtually connected to satellite centers, pooling expertise without relocating patients or surgeons. In the future, even emergency surgical interventions may be attempted remotely, delivering timely care in critical scenarios, reducing healthcare costs, and improving access to expertise [7].

A key challenge will be the continuous monitoring and maintenance of latency throughout the procedure. Ensuring consistently low latency and minimal jitter will be essential for the safe execution of telesurgery [8].

Broader implementation will also require addressing medicolegal and ethical issues, including ownership of surgical outcomes across jurisdictions [9].

Additionally, logistical aspects such as billing and inter-hospital insurance reimbursements must be standardized and accepted by healthcare networks for widespread adoption [10].

Finally, the development of structured training and certification pathways for remote surgeons and local teams must be prioritized, standardized, and regularly audited. Initial clinical trials using a dual-console setup of the SSI Mantra system [3], where a clinical trial on five human patients was performed successfully, have demonstrated that telesurgery is feasible with low-latency connectivity.

## Conclusions

To our knowledge, the interhospital robotic telesurgery conducted between Dharan Hospital, Salem, and Prashanth Hospitals, Chennai, is the first of its kind in India and a major milestone in surgical innovation. Our experience demonstrates that with current 5G and fiber-optic technologies, hospital-to-hospital telesurgery in India is possible. Further data and clinical trials are required prior to widescale implementation. 

With adequate infrastructure, indigenous technology, and trained personnel, such collaborations can take us closer to redefining surgical accessibility, bridging geographic divides, and elevating standards of care worldwide.
